# Membrane roughness as a sensitive parameter reflecting the status of neuronal cells in response to chemical and nanoparticle treatments

**DOI:** 10.1186/s12951-016-0161-5

**Published:** 2016-01-29

**Authors:** Chia-Wei Lee, Lan-Ling Jang, Huei-Jyuan Pan, Yun-Ru Chen, Chih-Cheng Chen, Chau-Hwang Lee

**Affiliations:** Research Center for Applied Sciences, Academia Sinica, 128 Sec. 2, Academia Road, Nankang, Taipei, 11529 Taiwan; Genomics Research Center, Academia Sinica, Taipei, 11529 Taiwan; Institute of Biomedical Sciences, Academia Sinica, Taipei, 11529 Taiwan; Institute of Biophotonics, National Yang-Ming University, Taipei, 11221 Taiwan; Department of Physics, National Taiwan University, Taipei, 10617 Taiwan

**Keywords:** Optical profilometry, Neuroblastoma cell, Paclitaxel, Charged nanoparticle

## Abstract

**Background:**

Cell membranes exhibit abundant types of responses to external stimulations. Intuitively, membrane topography should be sensitive to changes of physical or chemical factors in the microenvironment. We employed the non-interferometric wide-field optical profilometry (NIWOP) technique to quantify the membrane roughness of living neuroblastoma cells under various treatments that could change the mechanical properties of the cells.

**Results:**

The membrane roughness was reduced as the neuroblastoma cell was treated with paclitaxel, which increases cellular stiffness by translocating microtubules toward the cell membranes. The treatment of positively charged gold nanoparticles (AuNPs) showed a similar effect. In contrast, the negatively charged AuNPs did not cause significant changes of the membrane roughness. We also checked the membrane roughness of fixed cells by using scanning electron microscopy (SEM) and confirmed that the membrane roughness could be regarded as a parameter reflecting cellular mechanical properties. Finally, we monitored the temporal variations of the membrane roughness under the treatment with a hypertonic solution (75 mM sucrose in the culture medium). The membrane roughness was increased within 1 h but returned to the original level after 2 h.

**Conclusions:**

The results in the present study suggest that the optical measurement on membrane roughness can be regarded as a label-free method to monitor the changes in cell mechanical properties or binding properties of nanoparticles on cell surface. Because the cells were left untouched during the measurement, further tests about cell viability or drug efficacy can be done on the same specimen. Membrane roughness could thus provide a quick screening for new chemical or physical treatments on neuronal cells.

## Background

Membranes of living cells exhibit many topographic features, such as ruffles, ripples, wave-like pattern propagations, and local stiffness variations. These topographic features depend heavily on the membrane properties as well as the configurations of cytoskeletons. In particular, the membrane biophysics plays essential roles in neuron physiology and pathology. For example, the fluidity of plasma membranes affects the processing of amyloid precursor proteins in neuron cells [1]. Lulevich et al. revealed that amyloid-β (Aβ), the key pathogenic protein of Alzheimer’s disease, increases the stiffness of mouse neuroblastoma cell N2a by using atomic force microscopy [2]. Pan et al. found that Aβ could reduce the membrane roughness of neuroblastoma, and electrical stimulations reversed this effect [3]. But the detailed mechanisms were not clear. Spedden et al. reported that the stabilization of microtubules increased the stiffness of membranes when neurons were treated with paclitaxel (Taxol) [4]. These previous studies showed that the mechanical properties of neuronal cell membranes are sensitive to external physical or chemical treatments.

Gold nanopartices (AuNPs) have been widely used in biomedical applications. Liao et al. demonstrated that negatively charged AuNPs decreased the cytotoxicity of Aβ on human neuroblastoma cells [5]. Ma et al. found that AuNPs of a 16 nm average diameter accelerated the aggregation of Aβ into short fibril bundles. Therefore AuNPs could have the potential to reduce the self-aggregation of Aβ fibrils [6]. Although the blood–brain barrier is considered as a great challenge in nanoparticle-based treatments, nanomaterials still possess high potential for reducing the toxicity of Aβ and other neural toxic peptides related to neurodegenerative diseases [7]. Therefore a simple method to detect the responses of neuronal cells to nanoparticle treatment is also very desirable.

In the present study, we measured membrane roughness of mouse neuroblastoma cell N2a under the treatment of Taxol. In this way we demonstrated that the decrease in the roughness represents the increase of membrane stiffness caused by microtubule translocation. Then we studied the effects of differently charged AuNPs on the membrane roughness of the N2a cells. Finally we conducted a time-lapse measurement of the temporal variation of membrane roughness induced by a hypertonic solution that reduced the membrane tension.

## Results

**Fig. 1:**
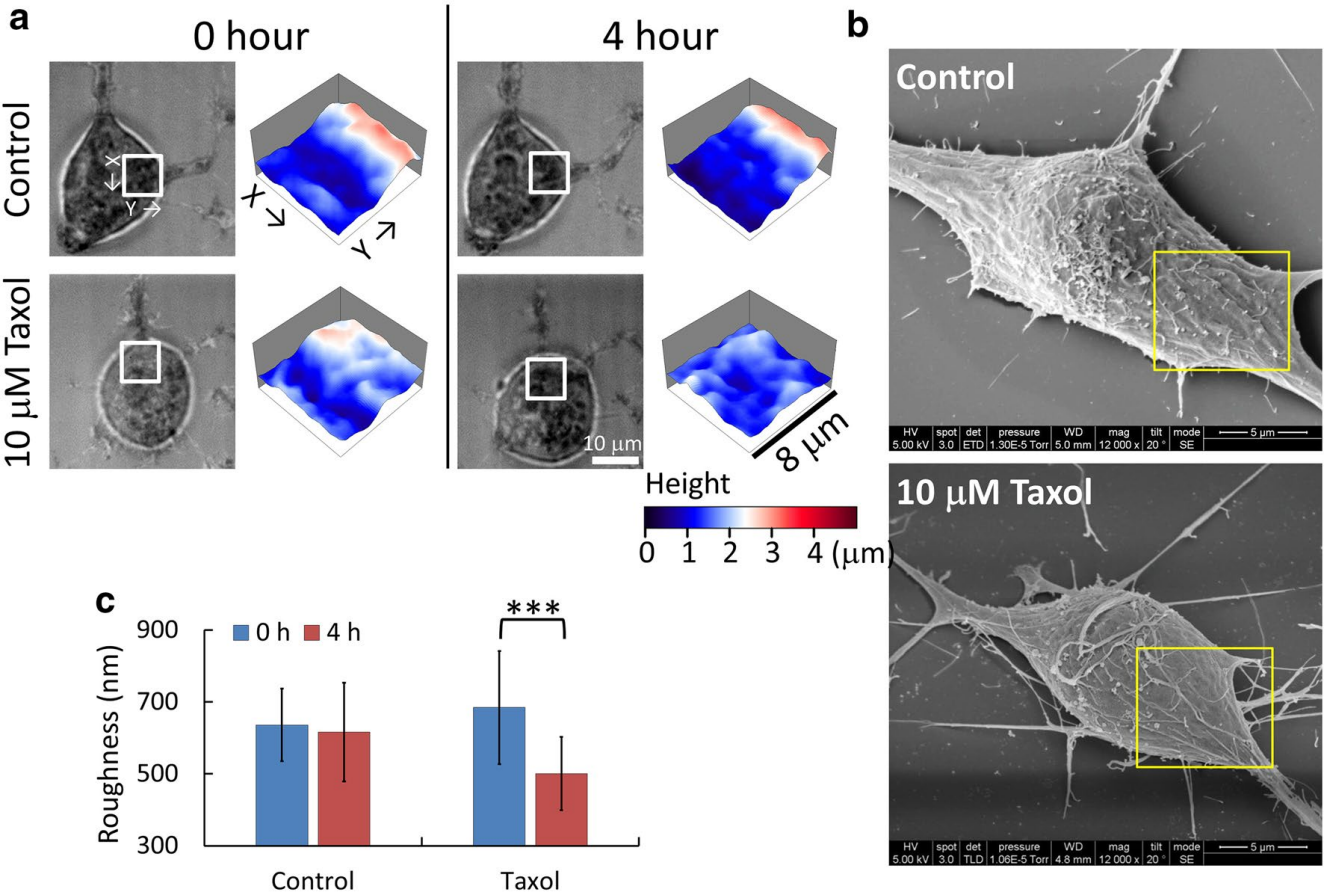
Variations of N2a cell membrane roughness under the treatment of Taxol. **a** Bright-field reflection image and the topography of N2a cells measured by NIWOP. Data was recorded without (control) or with the treatment of 10 μM Taxol. In each condition, the regions marked by the *white square* in the bright field images are displayed in the membrane topography. **b** SEM images of the N2a cells. The samples were measured after the treatment for 4 h. The membrane surfaces in the *yellow squares* show significant difference. **c** Statistics of N2a cell membrane roughness. The *blue bars* are measured before the treatment and the *red bars* are measured after 4 h of treatment. The cell number for the control and treatment group is 24 and 26, respectively. Data show the mean ± standard deviation. ***, *p* < 0.005 (Student’s *t* test)

We used the non-interferometric wide-field optical profilometry (NIWOP) system to measure membrane topography of living mouse neuroblastoma cells N2a. Spedden et al. had reported that the local stiffness of neuronal cells was increased in the areas near active neurites. The increase in cell stiffness was more related to the microtubule dynamics than the actin-filament structures [4]. Therefore we might use the measurement on membrane roughness to evaluate the effects of chemicals that influence the properties of microtubules. Taxol is a microtubule-stabilization drug. The treatment of Taxol alone on N2a cells does not induce neurite growth [8] but changes the mechanical properties of the cell. Therefore we are interested in how the membrane topography reflects this drug effect. Figure 1a shows the bright-field images and membrane topography measured by NIWOP of two N2a cells without and with the treatment of 10 μM Taxol. In the bright-field images, the cell morphology was not changed by Taxol. We compared the membrane roughness of an 8 × 8 μm^2^ region on the soma near a neurite, where the membrane activities were more obvious than other areas. The NIWOP topography showed that Taxol reduced the membrane roughness significantly. In order to confirm this optical observation, we also used scanning electron microscopy (SEM) to reveal the surface topography of fixed N2a cells. The ripple-like features on the cell surface were mostly removed by Taxol as shown in SEM micrographs (Fig. 1b). The cell surface became much smoother after the treatment. However, there was much cellular debris in the SEM micrographs. We suspected that the debris was produced during the cell fixation process. We repeated the NIWOP measurement on 26 cells to verify that the effect of Taxol treatment in reducing the membrane roughness of N2a cells (Fig. 1c). In other words, the stabilization of microtubules could lead to the smoothness of cell membranes.

**Fig. 2:**
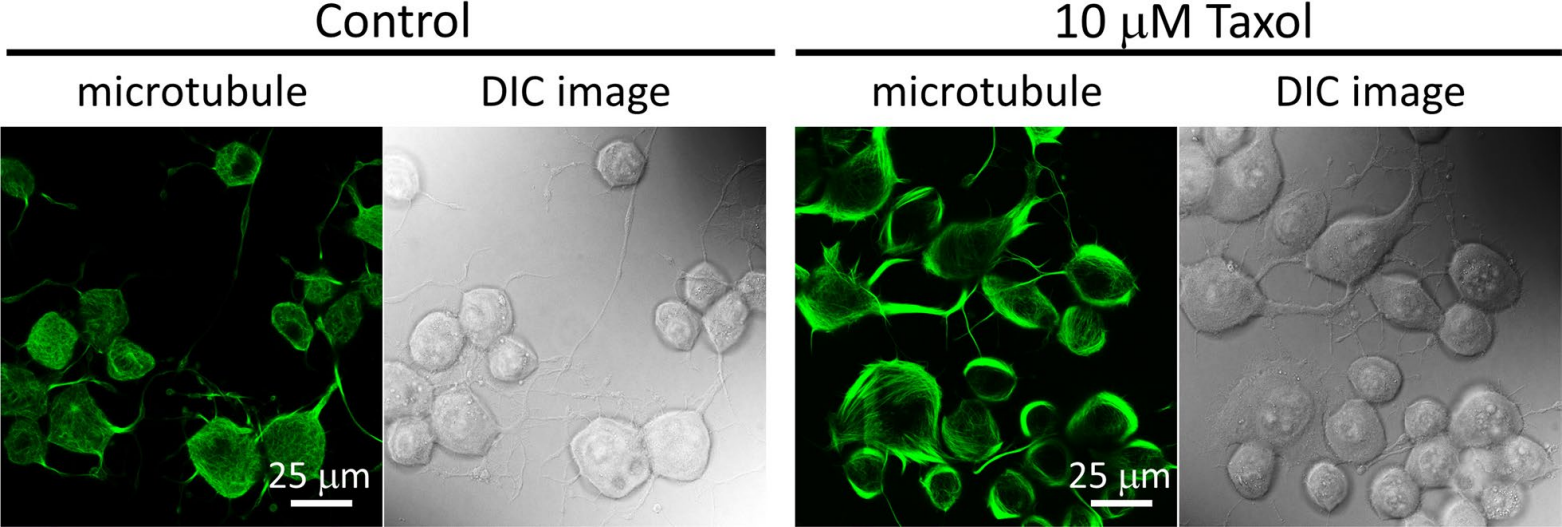
Confocal microscopy images of microtubules in N2a cells and differential interference contrast (DIC) images of the cells. The *panels* to the *right* show that the treatment of Taxol caused the redistribution of the microtubules toward the membranes

In order to reveal the effects of Taxol on microtubules, we used immunofluorescence images to observe the changes of microtubule distributions. Figure 2 shows that the treatment of 10 μM Taxol induced the redistribution of microtubules toward the cell membranes. Therefore, we suspected that the local membrane stiffness could be increased by Taxol. Combined with the measurement on membrane roughness, we suggest that the decrease in membrane roughness correlate with the increase in membrane stiffness.

**Fig. 3:**
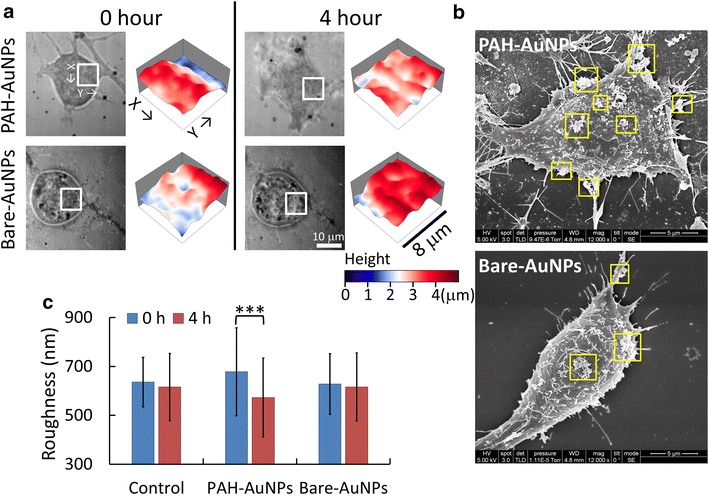
Variations of N2a cell membrane roughness under the treatment of AuNPs. **a** Bright-field reflection image and the topography of N2a cells measured by NIWOP. Data was recorded before and after 4 h of treatments. In each condition, the regions marked by the *white square* in the bright field images are displayed in the membrane topography. **b** SEM images of the N2a cells. The samples were measured after the treatment for 4 h. The *yellow squares* mark large aggregations of the AuNPs. **c** Statistics of N2a cell membrane roughness. The *blue bars* are measured before the treatment and the *red bars* are measured after 4 h of treatment. The cell number for the PAH-AuNPs and Bare-AuNPs group is 23 and 25, respectively. Data show the mean ± standard deviation. ***, *p* < 0.005 (Student’s *t* test)

Next, we used AuNPs as the treatment to test if membrane roughness also reflected the change of membrane properties induced by nanoparticle binding. Because cell membranes are negatively charged, nanoparticles of the same material but different charges could have different adsorption capabilities on cell membranes. Here we compared the effects of differently charged 30 nm AuNPs on membrane roughness of N2a cells. The bare AuNPs were negatively charged (zeta potential *V*_*ζ*_ = −14.3 mV, measured in the serum-free medium), while the AuNPs modified with poly-allylamine hydrochloride (PAH) were positively charged (*V*_*ζ*_ = 10.4 mV). Figure 3a shows that the positively charged AuNPs elevated the membrane topography uniformly, while the membrane roughness was reduced. However, for the 23 cells we observed in this experiment, the positively charged AuNPs did not always cause cell inflation. The morphological variations were quite random for individual cells. Therefore we could only conclude that the membrane roughness was reduced by the positively charged AuNPs. On the other hand, the negatively charged bare AuNPs did not cause significant changes in membrane roughness as well as cell morphology. The SEM micrographs in Fig. 3b show that the amount of adsorbed positively charged AuNPs was much more than that of the negatively charged AuNPs. The statistics of NIWOP measurements shown in Fig. 3c reveals that only the positively charged PAH-AuNPs reduced the membrane roughness of the N2a cells. The negatively charged bare AuNPs did not cause measurable change in the membrane roughness. Therefore, the membrane roughness of N2a cells could reflect the adsorption capability of nanoparticles onto the live cells.

**Fig. 4:**
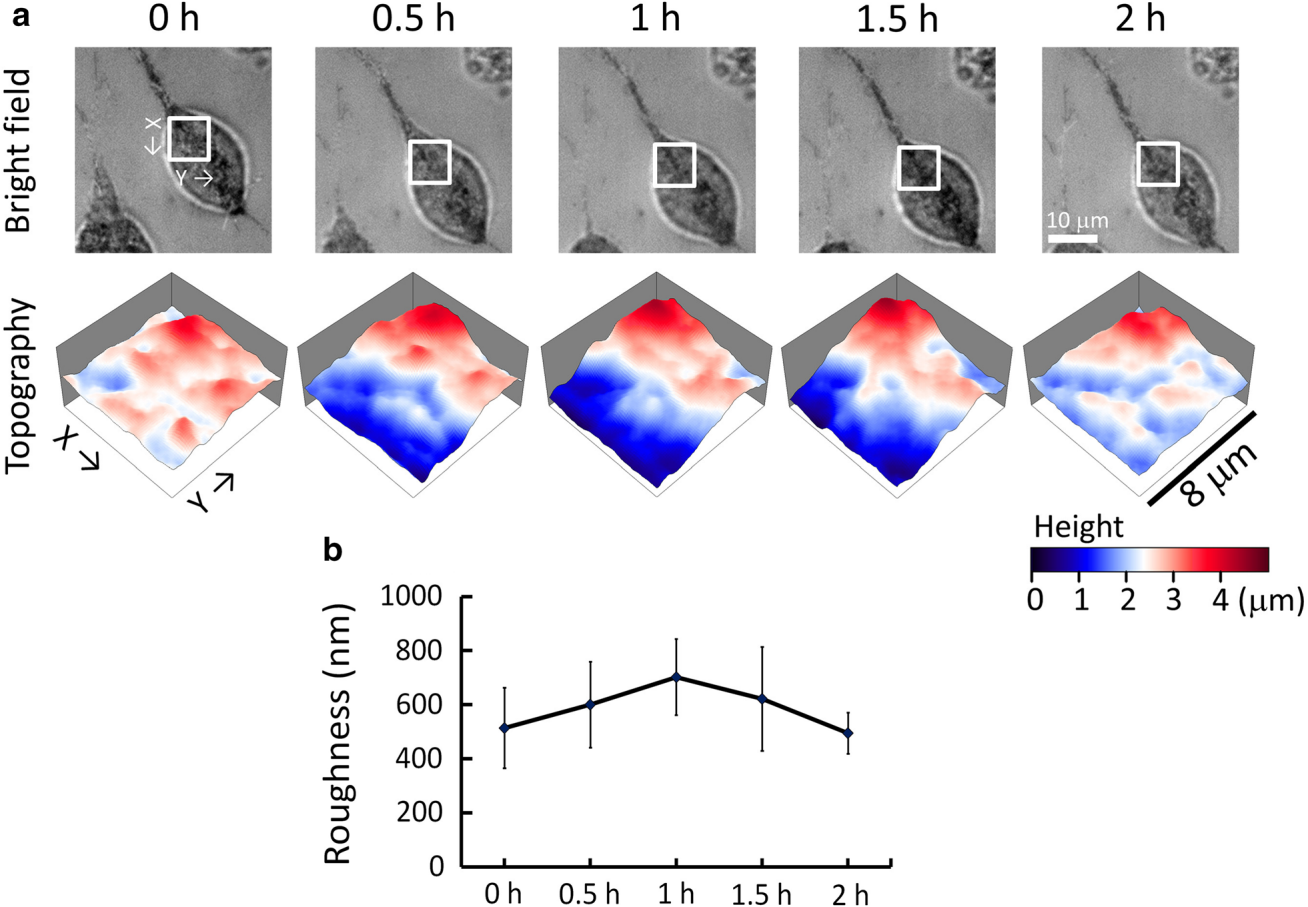
Variations of N2a cell membrane roughness induced by a hypertonic solution (75 mM sucrose in the culture medium). **a** The bright field images and topography of N2a cell membranes measured by NIWOP. Data was recorded at different time points after the treatment. In each frame, the region marked by the *white square* in the bright field image is displayed in the membrane topography. **b** Temporal variation of membrane roughness. Data are obtained from three independent experiments. *Error bar*, standard deviation

The optical measurement technique is particularly useful for time-lapse observations. Here we used a hypertonic solution (75 mM sucrose in the culture medium) to treat N2a cells and measured the temporal variation of membrane roughness. Because sucrose cannot penetrate cell membranes, high-concentration sucrose solution has been used as hypertonic buffer that reduces membrane tension without other metabolic effects in cells [9–11]. We suspected that the lower membrane tension might lead to an increase of the membrane roughness. Nevertheless, it was unknown how long the effect of a hypertonic solution on the membrane tension could sustain. Figure 4a shows the time-lapse bright-field images and NIWOP membrane topography of an N2a cell in the 75 mM sucrose solution, and the temporal variation of the membrane roughness is shown in Fig. 4b. The decrease of membrane tension resulted in an increase in membrane roughness in 1 h. Nonetheless, after 2 h the membrane roughness returned to the original level. The cell morphology was not changed during this observation period. This temporal variation of membrane roughness suggested that the effect of the hypertonic solution on the membrane tension might be transient because the cell could adapt the intracellular osmotic pressure to compensate this environmental stress.

## Discussion

In recent years, membrane roughness has been noticed as a sensitive cellular feature related to various stimulations, including cytoskeletal alteration [12], blood toxicants [13], anti-cancer chemicals or nanoparticles [14, 15], proteins related to neurodegenerative diseases [16], etc. In these works, the membrane roughness was obtained from AFM measurements, and therefore the results could be relevant to the membrane stiffness as well as the cytoskeletal organizations. Because the scanning force of the AFM tip is on the order of nanonewtons, and the image acquisition time is several minutes for one cell, the membrane roughness obtained with AFM could be regarded as a quasi-static mechanical property of the membrane–cytoskeleton complex in a cell.

In the present work we employed NIWOP, an optical profilometry using an ordinary objective as the probe, to obtain cell membrane roughness. Therefore, the measured membrane topography could be considered as a cellular feature under least mechanical perturbations. This is very different from the membrane topography obtained by AFM. Because a NIWOP frame was taken within 5 s, the measured membrane topography could represent the degrees of membrane undulations. Although the membrane roughness variations induced by the alteration of cytoskeletons, such as the microtubule translocation caused by the treatment of Taxol, was measurable, the optical measurement could be more sensitive to the membrane-associated stimulations (e.g., aggregation of Aβ on cell membranes), as we demonstrated in Ref. [3]. Recently it was reported that the aggregation of Aβ precursor protein on cell membranes could be important for the production of Aβ peptides [17]. If such activities of Aβ precursor protein on cell membranes also influence membrane mechanical properties, the measurement on membrane roughness could be used as a label-free assay on this issue. In addition, Aβ and ganglioside GM1 interactions resulting in formation of seeding templates on the membrane rafts may also affect cellular membrane roughness [18].

The membrane roughness is also sensitive to the adsorbability of nanoparticles on cell membranes. Considering the potential applications of various nanomaterials in the therapeutics of neuronal diseases [7], a simple and fast assay about the cellular responses to these nanoparticles could be very useful. In addition, because the NIWOP is a bright-field imaging technique, we might also include fluorescent markers or Raman spectroscopy to further exploring the cellular status under the treatment of nanomaterials in a single optical microscope.

Many cellular responses to external stimulations are transient. The time-lapse measurement on membrane roughness could be used to estimate the cellular adaptation rate to environmental variations. In the present work we changed the osmotic pressure of the culture medium by using 75 mM sucrose. Although this treatment reduced the membrane tension and increased the membrane roughness by nearly 1.5-folds, the membrane roughness returned to its original magnitude after 2 h. How the N2a cell adapts itself to compensate the change in the osmotic pressure is an intriguing question to investigate.

Optical profiling techniques with nanometre height accuracy are suitable for living cell characterizations. In addition to the NIWOP technique, membrane roughness might also be acquired by high-speed interferometric imaging techniques, such as live cell interferometry [19] or phase-shifted laser-feedback interference microscopy [20]. To decipher the variations of membrane roughness in response to stimulations in the microenvironment should also be useful in studying other cell activities, such as stem cell differentiation under the influences of substrate nanofeatures [21].

## Conclusions

In the present work we demonstrated that optical measurement on membrane roughness of neuronal cells could be a sensitive and fast diagnostic technique to reveal the cellular responses to external stimulations. Considering that the membrane physical properties of a neuronal cell play essential roles in neuron degenerative diseases, the membrane roughness can be employed as a quick test of cellular responses to potential drugs and nanomaterial treatments. Because the NIWOP technique is based on bright-field imaging, other optical contrast mechanisms such fluorescence or Raman scattering can be included into this assay for revealing relevant molecular mechanisms.

## Methods

### Setup of the non-interferometric wide-field optical profilometry (NIWOP)

The NIWOP technique [22] combines the concepts of differential confocal microscopy and wide-field optically sectioning microscopy. We employed the structured-illumination method to produce optical sectioning using a wide-field microscope [23]. The dorsal surface of a cell was placed into the linear region of the axial response curve of the sectioning microscopy, where the intensity is linearly proportional to the height of the sample. Because of the low cytoplasm absorption to the visible light, we had to use a calibration procedure to remove the reflection signal from the bottom surface of the cell [24]. After proper calibrations, membrane topography of an adherent cell could be obtained routinely [25, 26]. In the present work, the membrane roughness was defined as the standard deviation of the measured membrane topography within an 8 × 8 μm^2^ area near a neurite. The details of the most recent setup of our NIWOP system can be found in Ref. [3]. The depth resolution and dynamic range of the NIWOP system were 52 nm and ~3 μm, respectively. The whole system was placed in a temperature-controlled microscope cage, which provided a constant-temperature environment (37 ± 1 °C) for the live-cell experiments.

### Cell preparation

We used the cells of a mouse neuroblastoma cell line N2a as the samples in this work. The N2a cell line was obtained from Bioresource Collection and Research Center (Hsinchu, Taiwan). The cells were cultured in Minimum Essential Medium Alpha (MEM-α) (12,571, Gibco, Life Technologies, NY, USA) with 10 % fetal bovine serum and 1 % antibiotic pen-strep-ampho. For long-term observations, the cells were placed into a 100-mm culture dish and the culture area was sealed by a 0.17-mm-thick coverslip surrounded by double-sided adhesive tapes. The volume of the cell region was about 44.5 × 2.5 × 0.07 mm^3^. With this culture chamber the cells could be kept alive for more than 8 h in the temperature-controlled microscope cage. Before the treatment experiments of Taxol and AuNPs, the medium was replaced with the serum-free MEM-α.

For the observation of microtubules, the cells were fixed with 3.7 % formaldehyde in phosphate-buffered saline (PBS) for 30 min. Then the cells were permeabilized with 0.1 % Triton X-100 in PBS for 5 min and blocked with 1 % bovine serum albumin in PBS overnight. We used the anti-α tubulin antibody conjugate with Alexa Fluor^®^ 488 (Abcam, Cambridge, UK) to label the microtubules in the fixed N2a cells. The fluorescence images were acquired by a confocal microscope (TCS-SP5, Leica Microsystems, Wetzlar, Germany) with a 63×, 1.4 numerical aperture oil-immersion objective.

### Scanning electron microscopy (SEM) imaging

We employed a field-emission scanning electron microscope (Nova NanoSEM 200, FEI Company Corp., Hillsboro, OR, USA) to observe the membrane topography on fixed N2a cells. The cells were fixed with 2.5 % glutaraldehyde for 30 min and then washed twice with PBS. The water inside the fixed cells was replaced with ethanol (99.9 %) by gradually increasing the ethanol concentration. The fixed cells were then dried by using a critical-point dryer (EM CPD300, Leica Microsystems, Wetzlar, Germany). The dried fixed cells were coated with 10 nm Au film for better conductance required by SEM imaging.

### Gold nanoparticle (AuNP) preparation

The 30 nm AuNPs were purchased from Nanopartz Inc. (Loveland, CO, USA). The surface of the particles carries citrate anions as the capping agents during fabrication, and therefore these particles bear negative charges. We used them as the bare AuNPs in the present work without further modifications. In order to make positively charged AuNPs, the citrate-capped AuNPs were centrifuged at 5000 rpm for 5 min to remove the excess citrate. Then the AuNPs were re-dispersed with 200 μL de-ionized water and then added into 1 mL of 0.1 wt% poly-allylamine hydrochloride (PAH, molecular weight ~15,000, Sigma-Aldrich Corp., St. Louis, MO, USA) aqueous solution. The mixture was incubated overnight, centrifuged at 5000 rpm for 5 min, and then washed with de-ionized water. This procedure was repeated twice. The PAH-coated AuNPs were re-suspended in water and added into the culture medium for cell treatments.

We used NanoBrook 90Plus Zeta Particle Size Analyzer (Brookhaven Instruments Corp., Holtsville, NY, USA) to measure the zeta potentials and hydrated radii of the AuNPs. In the serum-free medium, the average hydrated radius of the bare AuNPs was 188 nm, while that of the PAH-coated AuNPs was 263 nm.
